# Exosomal miRNA-21 from *Toxoplasma gondii*-infected microglial cells induces the growth of U87 glioma cells by inhibiting tumor suppressor genes

**DOI:** 10.1038/s41598-022-20281-w

**Published:** 2022-09-30

**Authors:** Bong-Kwang Jung, Hyemi Song, Hyejoo Shin, Jong-Yil Chai

**Affiliations:** 1 MediCheck Research Institute, Korea Association of Health Promotion, Seoul, 07649 Korea; 2 Department of Tropical Medicine and Parasitology, Seoul National University College of Medicine, Seoul, 03080 Korea

**Keywords:** Microbiology, Molecular medicine

## Abstract

*Toxoplasma gondii* is an intracellular protozoan parasite that can modulate the microenvironment of infected hosts and is known to be associated with the incidence of brain tumor growth. In this study, we suggested that the exosomal microRNA-21 derived from *Toxoplasma* infection would contribute to the growth of brain tumors. Exosomes of BV2 microglial cells infected with *Toxoplasma* were characterized and confirmed internalization to U87 glioma cells. Exosomal miRNA expression profiles were analyzed using microRNA array and miR-21A-5p associated with *Toxoplasma* and tumor sorted. We also examined the mRNA level of tumor-associated genes in U87 glioma cells by changing the level of miR-21 within exosomes and the effects of exosomes on the proliferation of human U87 glioma cells. Expression of miRNA-21 was increased and anti-tumorigenic genes (FoxO1, PTEN, and PDCD4) were decreased in exosomes within *T. gondii*-infected U87 glioma cells. *Toxoplasma*-infected BV2-derived exosomes induced proliferation of U87 glioma cells. The exosomes induced the growth of U87 cells in a mouse tumor model. We suggest that the increased exosomal miR-21 from *Toxoplasma*-infected BV2 microglial cells may play an important role as a cell growth promotor of U87 glioma cells through a down-regulation of anti-tumorigenic genes.

## Introduction

More than 18.1 million common cancer cases were estimated to occur worldwide in 2018, and about 297,000 central nervous system tumors (1.6% of all tumors) are diagnosed every year^1^. Previous studies have found that risk factors for human brain tumors include various chemical products, family history, and ionizing radiation from therapeutic and diagnostic devices in the head^2^. However, the exact causes of these malignancies are yet unclear. Approximately 20% of various malignancies worldwide are due to infectious agents, including viruses, bacteria, and parasites^3,4^. Infectious agents can interfere with the host cell’s genetic types of machinery, such as DNA repair and cell cycle, and can lead to chronic inflammations and immune system impairments^5^.

The infectious agents associated with human cancers are most commonly viral pathogens, including the human papillomavirus and hepatitis B and C viruses^3^. Parasites may also play potential roles in the development of human cancers^6^. Several parasite species, namely, *Schistosoma haematobium*, *Opisthorchis viverrini*, *O. felineus, Clonorchis sinensis*, and *Hymenolepis nana* are related to the development of various types of human cancers^6–8^.

*Toxoplasma gondii* is an intracellular protozoan that can modulate the microenvironment of the infected host cells^9^. This parasite was estimated to infect about 30% of people worldwide, and the entire global population is at risk^9,10^. *Toxoplasma* can invade vital organs, including the central nervous system (CNS), and may cause severe diseases, such as fatal meningitis and encephalitis, particularly in immunocompromised patients^9^. However, *Toxoplasma* can also change the environment of infected hosts to maintain asymptomatic chronic infection through modulation of the cell growth and immune responses in immunocompetent individuals^9,11^. Interestingly, several reports suggested that altered environments in the host body due to chronic *Toxoplasma* infection are analogous to the tumor microenvironment, considering the correlation between *Toxoplasma* prevalence and brain tumor incidence^12–15^.

The exosomes are known as cell-to-cell communicators that deliver biological contents, including proteins and nucleic acids, from neighboring cells^16,17^. The exosomes can influence the tumor-related biological processes, such as anti-apoptosis, angiogenesis, and metastasis within the tumor microenvironment^18^. Especially, microRNAs (miRNAs) are small non-coding RNAs with ~ 22 nucleotides and are important post-transcriptional gene regulators controlling more than 30% of human mRNAs by miRNA-induced silencing complex (miRISC)^19^. *Toxoplasma* can subvert the biological processes by controlling miRNA expression in the infected hosts^20^. Host miRNAs have important clues to modulating host biological processes for the survival strategy of parasites. Therefore, the study of host miRNA profile changes in *Toxoplasma* infection can help us to understand the interaction between the host and *Toxoplasma* more clearly. Actually, Thirugnanam et al.^15^ hypothesized that *Toxoplasma* promotes brain carcinogenesis by changing its expression on specific host miRNAs related to tumor growth and revealed that *Toxoplasma* could cause glioma in experimental animals.

The present study focused on the alteration of exosomal miR-21 in *Toxoplasma*-infected host BV2 microglial cells. We observed the possible role of altered exosomal miR-21 in the growth of U87 glioma cells through the nuclear retention of FoxO1/p27 targeted by overexpressed miR-21.

## Results

### Characterization of microglial cell-derived exosomes

**Figure 1:**
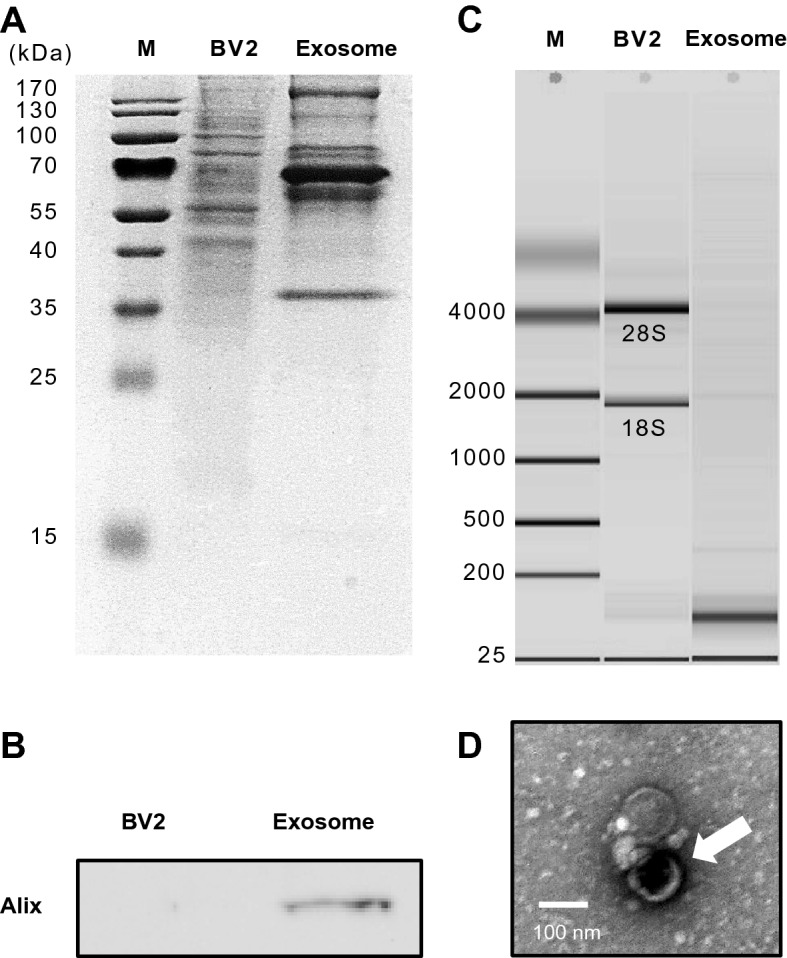
Characterization of BV2 cell-derived exosomes. (**A**) SDS-PAGE. Protein was isolated from either BV2 cells or BV2-derived exosomes. The pattern of proteins was different between the cell and exosomes. (**B**) Western blot analysis of exosome marker (Alix). (**C**) Purified RNA from BV2 cells and BV2-derived exosomes were evaluated using a Bioanalyzer. As a result, the 18S and 28S ribosomal subunits in BV2 cells were rarely observed in the RNA of exosomes. (**D**) Transmission electron microscopy showing an exosome isolated from BV2 cells stained negatively with 2% uranyl acetate. The exosomes were approximately 60–150 nm in size with a cup-shaped structure (Song and Jung, unpublished data).

BV2-derived exosomes were achieved using differential centrifugation and verified by various methods to prevent contamination with cellular components or other vesicles. SDS-polyacrylamide gel electrophoresis (SDS-PAGE) showed different patterns between extracted proteins from BV2 cells and exosomes (Fig. 1A), and the samples were evaluated for the presence of Alix, an exosomal protein marker in western blot analysis. Alix marker was detected in the exosomal proteins but not in the BV2 cell lysate proteins (Fig. 1B). In addition, purified RNA from BV2-derived exosomes was analyzed by a bioanalyzer. The 18S and 28S nuclear ribosomal subunits were rarely observed in the RNA migration patterns of exosomal RNA, indicating that the purity was reliable (Fig. 1C). Finally, transmission electron microscopy revealed that the observed exosomes were shown in approximately 60–150 nm sizes with a cup-shaped structure which is a typical exosome morphology (Fig. 1D).

### *Toxoplasma*-infected BV2-derived exosomes induced proliferation and anti-apoptosis of glioma cells in vitro

**Figure 2:**
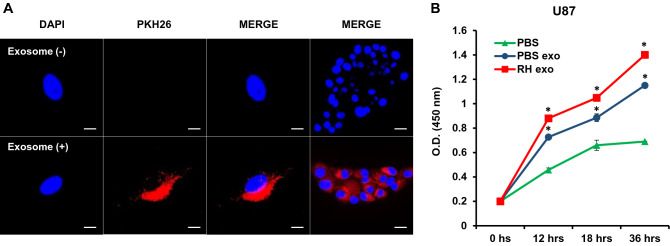
Cellular internalization of BV2-derived exosomes into U87 glioma cells and the proliferation of U87 glioma cells induced by Toxoplasma RH-infected BV2-erived exosomes. (**A**) Exosomes taken up by U87 cells as determined by confocal microscopy. U87 glioma cells were incubated with either PKH26-labeled exosomes (red) or without as control for 24 h. The nuclei of cells were stained with DAPI (blue) before observing in confocal microscopy (scale bars: 10 μm, × 3000). (**B**) The proliferation of U87 glioma cells was determined by a cell proliferation assay. U87 glioma cells were treated with exosomes for the indicated times. *P < 0.05 was obtained by Student’s *t* test.

The cellular internalization of BV2-derived exosomes into U87 human glioma cells was visualized by confocal microscopy. PKH26-labeled exosomes were localized in the cytoplasm of U87 cells. The nuclei were stained with DAPI (Fig. 2A), implying that the BV2-derived exosomes could be internalized by host cells and would influence the environment of recipient cells.

After confirming the cellular internalization of BV2-derived exosomes into U87 glioma cells, we performed a cell proliferation assay to investigate the role of *Toxoplasma*-infected BV2 cells-derived exosomes in human glioma cell development. U87 cells were treated with exosomes of *Toxoplasma*-infected BV2 cells, and the results indicated that *Toxoplasma*-infected BV2-derived exosomes induced a significantly higher proliferation of U87 cells compared to controls (Fig. 2B).

Also, the growth of U118 cells has the same consequence as U87 in that *Toxoplasma*-stimulated exosomes caused the highest level of proliferation (data not shown). Based on these data, we could represent that *Toxoplasma*-infected BV2-derived exosomes play an essential role in the proliferation of glioma cells.

### *Toxoplasma*-infected BV2-derived exosomes induce the growth of tumors in vivo

For investigating the effect of *Toxoplasma*-infected BV2-derived exosomes in tumor development, we injected U87 glioma cells into nude mice for a xenograft model and administrated BV2-derived exosomes or RH-infected BV2-derived exosomes to the mice. Once the tumor was apparent after 1 week, each experimental group, consisting of five mice, was divided based on tumor size for defining the same starting point, and tumor size was measured for 22 days.

**Figure 3:**
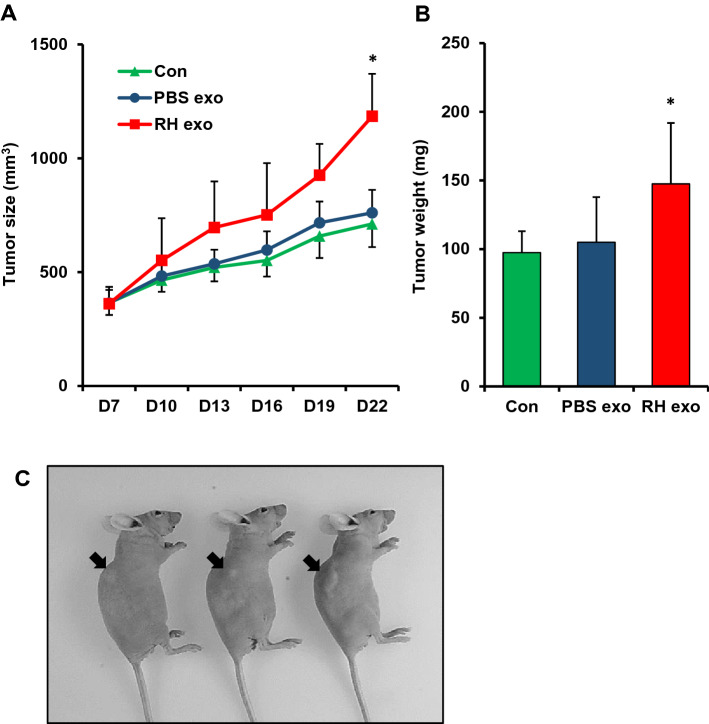
Tumorigenic effects of BV2-derived exosomes in U87 xenograft mouse model (**A**–**C**). Significantly increased tumor size (**A**) and weight (**B**) of BALB/c nude mice treated with RH-infected BV2-derived exosomes. BALB/c nude mice (**C**) were subcutaneously injected with 1 × 10^7^ U87 cells suspended in a Matrigel mixture. After 6 days post-injection, 100 µg of BV2-derived exosomes were treated in the mice. Tumor size and weight were measured on the indicated days and after sacrifice, respectively. *P < 0.05.

In the U87 xenograft model mice, significantly higher tumor size and weight were observed in the RH-infected BV2-derived exosome group on day 22 (Fig. 3A,B). On the other hand, both the BV2-derived exosome group and the control group had no significant differences in the tumor size by treatment with exosomes. Also, the mice injected with glioma cells and exosomes exhibited the largest tumor volume visually in the RH-infected BV2-derived exosome group (Fig. 3C). These results demonstrated that *Toxoplasma*-infected BV2-derived exosomes induced glioma growth in a mouse tumor model.

### Expression changes of exosomal miR-21a-5p (miR-21) in microglial cells infected with *Toxoplasma* (RH)

**Figure 4:**
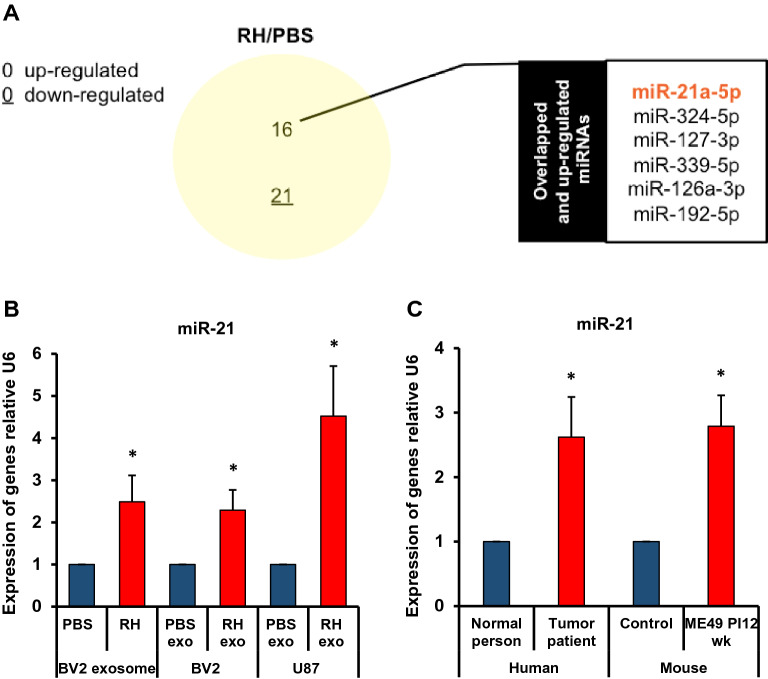
Expression changes of exosomal miR-21a-5p in microglial cells with *Toxoplasma* (RH) infection. (**A**) It is shown that the immunity or tumor development-related miRNAs were significantly changed in those following the *Toxoplasma* RH infection. (**B**) The relative expression level of miR-21 was examined in the BV2-derived exosomes, exosomes treated with BV2, and U87 cells by real-time RT-PCR. (**C**) The relative expression level of miR-21 was examined in brain tissue from tumor patients (N = 3) and *Toxoplasma* (ME49 strain) infected mice (N = 3). *P < 0.05 was obtained by Student’s *t* test.

The data showed 37 miRNAs (16 up- and 21 down-regulated miRNAs) related to the immunity or tumor development were significantly changed in microglial cells after infection with *Toxoplasma* RH strain (Fig. 4A). The relative expression levels of miR-21 among altered miRNAs were validated in the BV2-derived exosomes, exosomes treated with BV2 and U87 cells by real-time RT-PCR. The expression of miR-21 exhibited a significant increase in exosomes of *Toxoplasma* (RH strain)-infected BV2 cells (Fig. 4B). The relative expression level of miR-21 in BV2 and U87 cells was increased after the uptake of altered exosomes (Fig. 4B). The relative expression level of miR-21 was higher in the brain tissue from tumor patients and *Toxoplasma* (ME49 strain) infected mice than in the control group, respectively (Fig. 4C). These results were correlated with changes between the expression levels with predicted and validated miRNAs in vitro and in vivo.

### *Toxoplasma*-infected BV2-derived exosomes down-regulate anti-tumorigenic target gene expressions in U87 cells

**Figure 5:**
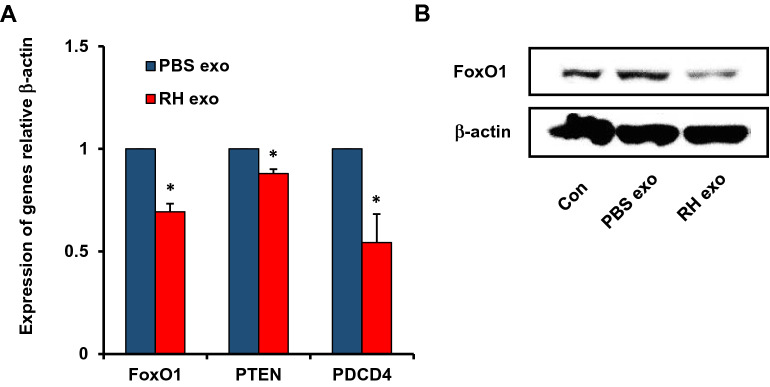
*Toxoplasma* RH-infected BV2-derived exosomes induce downregulation of anti-tumor associated genes in U87 glioma cells by *Toxoplasma* RH-infected BV2-derived exosomes. (**A**) Real-time PCR for FoxO1, PTEN, and PDCD4 expressions in *Toxoplasma* RH-infected BV2-derived exosomes versus those with PBS exosomes. β-actin mRNA was used as a control. (**B**) FoxO1 expression was determined by Western blot analysis and the statistical evaluation of densitometry data was performed using the ImageJ program. *P < 0.05 was obtained by Student’s *t* test.

Exosomes derived from RH-infected BV2 cells led to the growth of glioma in vivo and in vitro (Figs. 2, 3). In order to discover the related mRNA, we examined the mRNA levels of anti-tumorigenic target genes, Fork-head box O1 (FoxO1), PTEN, and programmed cell death 4 (PDCD4) in U87 cells treated with BV2- or RH-infected BV2-derived exosomes. The bioinformatics analysis suggested that multiple tumor-related genes, including FoxO1, PTEN, and PDCD4 genes, have miR-21 binding sites^21,22^. The mRNA levels of anti-tumorigenic target genes were decreased in RH-infected BV2-derived exosomes compared to BV2-derived exosomes (Fig. 5A). The FoxO1 showed a reduced protein level in RH-infected BV2-derived exosomes compared to BV2-derived exosomes (Fig. 5B). From these results, we could confirm that the down-regulated anti-tumorigenic genes by RH-infected BV2-derived exosomes support its role in tumor growth.

### Changed expression of FoxO1 targeted by RH-infected BV2-derived exosomal miR-21 in U87 glioma cells

**Figure 6:**
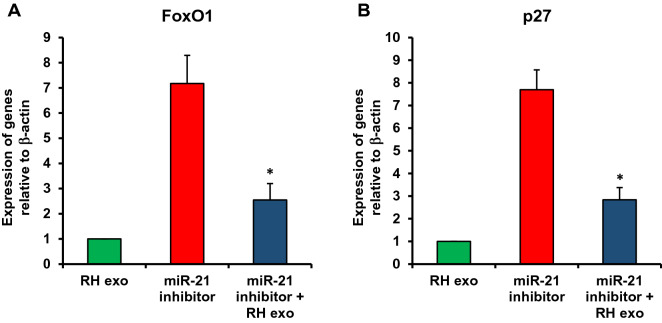
Changed expression of FoxO1/p27 targeted by RH-infected BV2-derived exosomal miR-21 in U87 glioma cell. U87 cells were transfected with a miR-21 inhibitor using Lipofectamine 2000, and the cells were harvested 24 h after transfection. The expression levels of FoxO1 and p27 in cells transfected with the miR-21 inhibitor were compared with those treated with BV2-derived exosomes using qRT-PCR (**A**,**B**).

In order to know the effect of miR-21 in exosomes on tumor-related gene regulation, U87 cells were transfected with a miR-21 inhibitor using Lipofectamine 2000, and the cells were harvested 24 h after transfection. The expression levels of FoxO1 and p27 in cells transfected with the miR-21 inhibitor were compared with those treated with BV2-derived exosomes using qRT-PCR (Fig. 6A,B). The miR-21 inhibitor transfection in U87 cells significantly suppressed the FoxO1 and p27 expression (Fig. 6).

## Discussion

To evade from host immune responses, *Toxoplasma* parasites transform themselves into tissue cysts. They are parasitic on various tissues, including the brain, heart, and skeletal muscle for the lifetime of the host, modulating the host immune responses^23^. Furthermore, they can also modulate the cell cycle and apoptosis of the host cells for their proliferation^14,24^. *Toxoplasma* preferentially infects host dendritic cells, neutrophils, and monocyte/macrophage lineage cells, including microglia in the brain^25^. *Toxoplasma* induces the differentiation of M2 phenotype macrophages, which influence wound repair after pathogen infections and is also associated with a high degree of vascularization and granuloma fibrosis^26^. Such behavioral pathogenesis of *Toxoplasma* infection may be linked to the related hallmarks of tumor development. The unfavorable environment modulated by *Toxoplasma* may be similar to appropriate precancerous conditions. Thus, we can presume that *Toxoplasma* infection should help develop brain tumors. Actually, it was reported that various brain tumor patients showed a high prevalence of *Toxoplasma* infection in sera^13^. Additionally, *Toxoplasma* can be another oncogenic effector and synergistically act as a helper of other infectious carcinogens developing the brain tumor. In this respect, it is noteworthy that *Plasmodium falciparum* and Epstein–Barr virus contributed synergistically to the formation of Burkitt’s lymphoma^27^.

There are comprehensive studies on the roles of exosomes as a regulator in the cancer research area. However, the roles of exosomes between parasites and infected hosts are still poorly understood. So far, various modulating factors, including secretory proteins have explained the biological process of protozoan parasites to survive against host attacks and establish infection^16^. Recently, there has been an increasing concept that protozoa-associated microvesicles and their miRNAs communicate with host cells for promoting a favorable environment for their survival^28^. Thus, further studies are needed to find the relationship between altered exosomal miRNAs and the proliferation of glioma cells. The changing miRNAs (miR-30c-1, miR-125b-2, miR-23b-27b-24-1, and miR-17–92 cluster genes) are regulated through promoter binding of the STAT3 in *Toxoplasma*-infected human macrophages and induced anti-apoptosis in response to *Toxoplasma* infection^29^. *Toxoplasma* infection can increase the expressions of miR-17-5p and miR-106b-5p, which have been implicated in several hyperproliferative diseases^30^. This evidence suggests that host miRNAs modulated by *Toxoplasma* infection are essential molecules in host biological behaviors for parasite survival and pathogenesis.

The altered miRNAs can affect various behaviors of the initiation and progression of malignant cells, including glioma; self-sufficiency in growth signals, insensitivity to anti-growth signals, evasion from apoptosis, limitless replicative potential, angiogenesis, invasion and metastasis, and inflammation^31^. In glioma, altered miRNAs have been identified by several expressed profiling studies^32^.

In the present study, we confirmed the high expression level of miRNA-21 in *Toxoplasma*-infected host cells. The miR-21 has been identified as one of the most commonly overexpressed miRNAs in solid tumors, including glioma^33^, and its expression correlates with glioma grade^34^. Accumulating evidence suggests that miR-21 is a novel oncogene as an anti-apoptotic factor in the growth of glioma^35^ and is strongly overexpressed in malignant human brain tumor tissue and plasma^36^. Interestingly, the inactivation of miR-21 in glioma cells and tissues triggers the inhibition of cell proliferation by caspase-dependent apoptosis^37^. The bioinformatics analysis of miR-21 predicted targets suggested that multiple tumor suppressor genes, including programmed cell death 4 (PDCD4), tropomyosin (TPM1), PTEN, and Fork-head box O1 (FoxO1) related to apoptosis pathways, have miR-21 binding sites^21,22,38^.

FoxO1, as one of the (FoxO) transcription factors, is involved in the development of various types of human cancers^39^ and can regulate tumor suppressor gene expression, such as p21, p27, Bim, and FasL^40^. The FoxO1 could bind and activate cell-cycle inhibitors, such as p27, to inhibit cell growth^41^. Moreover, FoxO1 is a crucial effector of PI3K/Akt signaling and regulates many biological processes, such as cell cycle progression and cell differentiation through the transcriptional activation of p27^42^.

**Figure 7:**
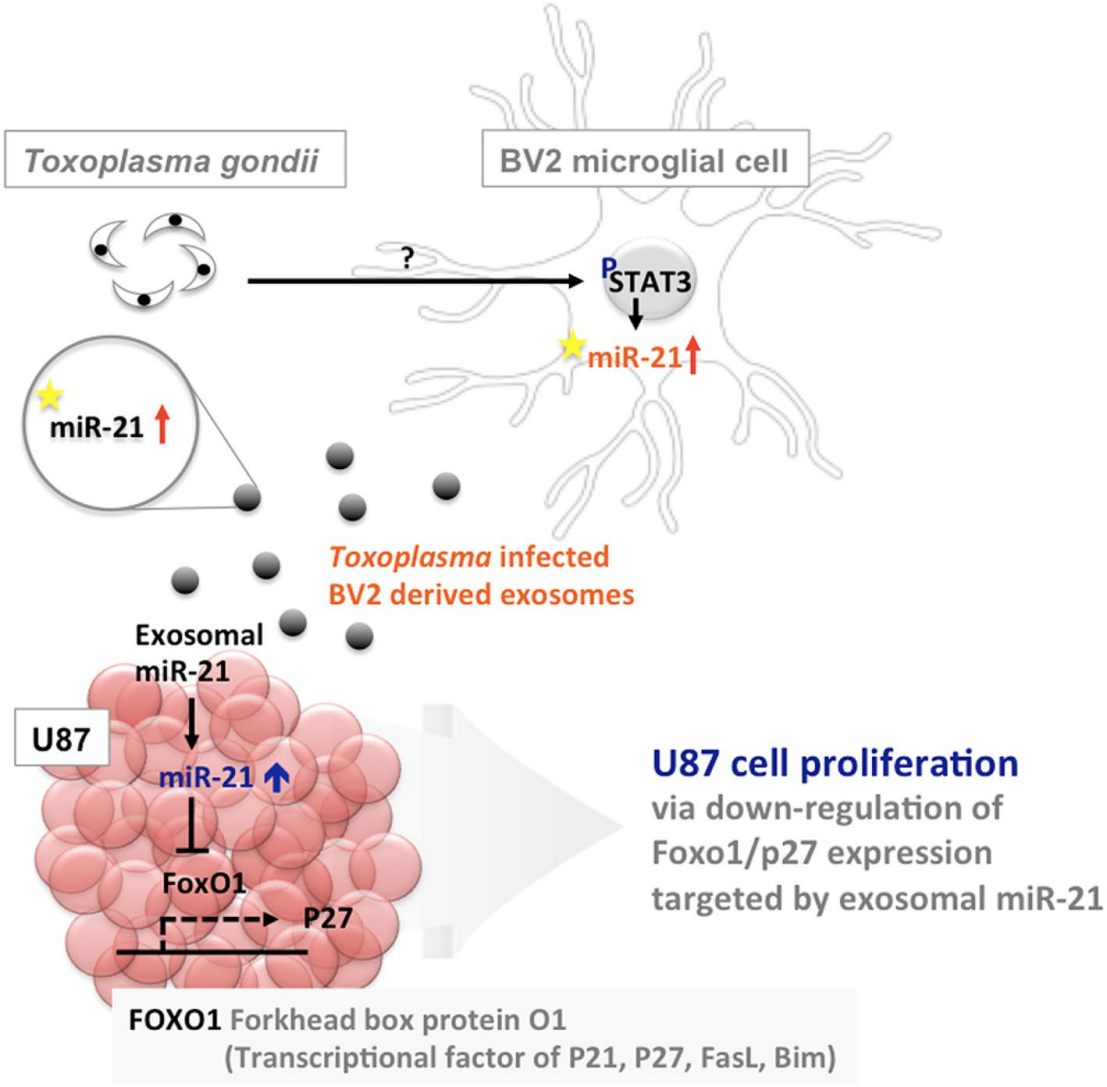
Schematic representation of carcinogenesis mechanisms of glioma (brain) suggested by the present study. Drawn by the authors using the PowerPoint 2019 version (Microsoft, Redmond, WA).

Conclusively, we suggest that *Toxoplasma*-infected microglia cell-derived exosomal miR-21 could play an essential role as a regulator for the growth of glioma cells (Fig. 7). However, further studies are needed to find a direct relation between changed exosomal miR-21 by *Toxoplasma* infection and glioma growth. These findings are expected to provide a starting point of research about the relationship between *Toxoplasma* infection and glioma incidence.

## Methods

### Ethics for animal experiments

All experimental protocols, including animal use, in this study followed the standard ethical guidelines of the Institutional Animal Care and User Committee at Seoul National University and have been approved by the Institutional Review Board of Seoul National University College of Medicine (IRB no. SNU-150715-2). All experimental procedures were carried out in compliance with the ARRIVE guidelines.

### Cell culture

BV2 murine microglial cells and U87 human glioma cells were cultured respectively in Dulbecco’s Modified Eagle’s Medium (DMEM; Welgene, Seoul, Korea) and Roswell Park Memorial Institute medium (RPMI; Welgene) each containing 10% fetal bovine serum, 4 mM l-glutamine, 0.2 mM penicillin, and 0.05 mM streptomycin. These cells were cultured at 37 °C in a 5% CO_2_ incubator. Another glioma cell line, U118, was used for comparison with U87 cells.

### *Toxoplasma gondii* infection

For the isolation of exosomes derived from the RH and ME49 strains of *T. gondii* infections, the tachyzoites of *Toxoplasma* (RH strain) were collected from the peritoneal cavity of 6-week-old BALB/c mice that had been injected 3–4 days previously. The tachyzoites were washed 3 times with phosphate-buffered saline and purified by centrifugation over 40% Percoll (Sigma-Aldrich, St. Louis, MO, USA)^43^. To prepare tachyzoites of the ME49 strain, 20 tissue cysts were intraperitoneally injected into BALB/c mice, and at day 6 to 8 post-infection (PI), the tachyzoites converted from the bradyzoites in the cysts were harvested by washing the peritoneal cavity of infected mice with PBS. The tachyzoites of ME49 were cultured in Vero cells (monkey kidney cells, KCLB cell line; no. 10081) grown in complete RPMI (Roswell Park Memorial Institute) 1640 media (WelGENE Inc., Daegu, Korea) supplemented with 100 μg/mL of penicillin (Gibco/BRL, Grand Island, NY, USA), 100 μg/mL of streptomycin (Gibco/BRL), and 5% fetal calf serum (Lonza, Walkersville, MD, USA) at 37 °C under an atmosphere of 5% CO_2_. After culturing in Vero cells, the tachyzoites of ME49 were passed through a 25-gauge needle twice, and then debris and cells were removed by passing through 5-μm filter membranes. After washing, the tachyzoites were re-suspended in PBS^44^. The tissue cyst of *T. gondii* ME49 strain was maintained by injecting cysts isolated from the brains of infected C57BL/6 mice (Orient Bio Animal Center, Seongnam, Korea) intraperitoneally. The brains of mice infected with ME49 were harvested at 3 months PI and minced to isolate cysts under a microscope. Infected mice were raised under specific pathogen-free (SPF) conditions at Seoul National University College of Medicine.

### RNA purification

Total RNA was extracted from BV2-derived exosomes, BV2 cells, and tissue using miRNeasy Mini Kit (Qiagen, Hilden, Germany) according to the manufacturer's instructions, including incubation time at the elution step. The RNA concentration was determined by NanoDrop 2000 Spectrophotometer. RNA quality for microarray was assessed by Agilent 2100 bioanalyzer (Agilent Technologies, Amstelveen, Netherlands).

### Exosomes isolation

DMEM with 10% exosome-depleted FBS was prepared by ultracentrifugation at 100,000*g* for 16 h at 4 °C with filtration through a 0.22-µm filter (Nalgene, Rochester, NY, USA). BV2 cells, 5 × 10^5^, were cultured in DMEM with 10% exosome-depleted FBS and 1% antibiotics at 37 °C and 5% CO_2_. After 24 h incubation, the tachyzoites of RH or ME49 strain (MOI = 10) were added to the cells, and non-invaded parasites were removed in an hour and refilled with the DMEM. Exosomes from BV2 cells were isolated by modified differential centrifugation, the most widely used method. The pellet consisting of exosomes was resuspended in 300 μL of PBS for RNA or protein analysis. The concentration of isolated exosome was determined by BCA protein assay kit (Pierce, Rockford, IL, USA) and NanoDrop 2000 Spectrophotometer.

### Characterization of exosomes

The pellet from either BV2 cells or BV2-derived exosomes was lysated in PRO-PREP™ protein extraction solution (iNtRon Biotechnology, Seongnam, Korea), and the protein was loaded on 10% SDS polyacrylamide gel stained with Coomassie brilliant blue. Also, the protein was transferred to PVDF membranes for 2 h. Western blotting was confirmed with Alix (Cell signaling Technology, Beverly, MA, USA) antibody as an exosome marker. HRP-conjugated goat anti-mouse IgG (H + L) (Bethyl Laboratories, Montgomery, TX, USA) antibody was used as a secondary antibody, and the immune-reactive band was visualized using a luminescent image analyzer LAS-1000 plus (Fuji Photo Film, Tokyo, Japan). Transmission electron microscopy was performed to examine the size and morphology of the exosomes. Exosomes (6.40 µg/µL) isolated from BV2 cells were prepared on a carbon-coating grid and negatively stained using 2% uranyl acetate for 1 min^45^. The prepared samples were observed using a JEOL 1200-EX II (Tokyo, Japan) equipped with an ES1000W Erlangshen CCD camera (Gatan, Pleasanton, CA, USA) at an accelerating voltage of 80 kV.

### Confocal microscopy

BV2-derived exosomes were stained using PKH26 Red Fluorescent Cell Linker Kit (Sigma-Aldrich, St. Louis, MO, USA) for 15 min at room temperature. U87 cells, 2 × 10^5^, were incubated with either PKH26-labeled exosomes (red) or without exosomes as a negative control for 24 h at 37 °C in a 5% CO_2_ incubator. The nuclei of U87 cells were stained with DAPI (blue), and U87 cells were fixed in 4% paraformaldehyde for 15 min at 4 °C before observing in Leica TCS SP8 STED CW confocal microscopy system (Leica Microsystems, Mannheim, Germany).

### Real-time RT-PCR

The cDNA was synthesized from miRNA with a Mir-X miRNA First Strand Synthesis and SYBR qRT-PCR Kit (Takara Bio Inc., Shiga, Japan). Quantitative real-time PCR with an iQ5 real-time PCR detection system (Bio-Rad, Hercules, CA, USA) used primers and templates mixed with the SYBR Premix. DNA was amplified for 40 cycles of denaturation for 15 s at 95 °C and annealing for 60 s at 60 °C. The data generated from each PCR reaction were analyzed using the Data Analysis Module by iQ™5 optical system software (Bio-Rad). The relative changes in gene expression were calculated between selected target genes and ß-actin/miRNAs (and U6) using the standard curve method. Primer sequences used are shown in Table 1.

**Table 1:** Primers used in this study.

Target gene	Primer	Sequence (5′–3′)
β-Actin	Forward	ACC TGC AGC AAT ACC ATT GAC
	Reverse	AAG GTG AGG GAC TCA AAC TGC
VEGF	Forward	TAC CTC CAC CAT GCC AAG TG
	Reverse	GAT GAT TCT GCC CTC CTC CTT
TPM1	Forward	CTC TCA ACG ATA TGA CTT CCA
	Reverse	TTT TTT TAG CTT ACA CAG TGT T
PDCD4	Forward	TAT GAT GTG GAG GAG GTG GAT GTG A
	Reverse	CCT TTC ATC CAA AGG CAA AAC TAC AC
mmu-miR-21a-5p (MIMAT0000530)		TAGCTTATCAGACTGATGTTGA

### Cell proliferation assay

U87 glioma cells, 3 × 10^4^, were seeded in a 96-well plate and incubated in 100 µl of the conditioned media with *Toxoplasma*-infected BV2-derived exosomes (50 µg/ml) or non-pulsed BV2-derived exosomes (50 µg/ml) as the control for 12, 18, and 36 h. Cell proliferation rates were determined using a Cell Counting Kit-8 (Dojindo, Kumamoto, Japan) (Supplementary Figs. S1–S3)^46^.

### In vivo xenograft model

Five-week-old female BALB/c nude mice were purchased from Orient Bio (Seongnam-Si, Korea), and mice were housed individually in sterile cages under a constant room temperature (22 ± 2 °C) and humidity (45 ± 15%) with a 12-h light and 12 h-dark cycles in SPF condition (Seoul National University College of Medicine Animal Center). Mice were randomly divided into three groups (5 mice a group), and all groups received subcutaneous injections of 400 ml PBS per mouse containing 1 × 10^7^ U87 glioma cells with growth factor-reduced BD Matrigel™ (BD Science, Miami, FL, USA). After 6 days of tumor injection, 200 mg of exosome derived from BV2 cells with/without *Toxoplasma* infection was injected into the tumor site. The tumor sizes of mice in the groups were measured with a caliper three times a week for 22 days after tumor challenges, and the tumor volume was calculated using the formula: 0.5 × (Width)^2^ × Length.

### miRNA microarray

The miRNA expression was analyzed with miRCURYTM LNA microRNA Array, 7th generation-has, mmu, and rno array (EXIQON, Vedbaek, Denmark), covering 1,119 well-characterized mouse microRNA among 3,100 capture probes for human, mouse, and rat miRNAs. In this procedure, 5′-phosphates from 250 to 1000 ng of total RNA were removed by treating Calf Intestinal Alkaline Phosphatase followed by labeling with Hy3 green fluorescent dye. Labeled samples were subsequently hybridized by loading onto a microarray slide using Hybridization Chamber Kit (Agilent Technologies, Santa Clara, CA, USA) and Hybridization Gasket Slide Kit (Agilent Technologies). Hybridization was performed over 16 h at 56 °C followed by washing the microarray slide as recommended by the manufacturer. Processed microarray slides were then scanned with Agilent G2565CA Microarray Scanner System (Agilent Technologies). Scanned images were imported by Agilent Feature Extraction software version 10.7.3.1 (Agilent Technologies), and fluorescence intensities of each image were quantified using the modified Exiqon protocol corresponding GAL files. The microarray data of the current study is deposited at the GEO database under accession number GPL32397.

### Altered microRNAs analysis

Expression profiling of mature exosomal miRNAs in microglial cells by the RH or ME49 strain of *Toxoplasma* infection was analyzed using various web tools. The miRNAs related to tumor development were identified using miRWalk2.0 (http://mirwalk.umm.uni-heidelberg.de) and filtered with more than 8.0 normalized signal intensity (log2). Among the miRNAs, differentially expressed miRNAs by greater than 1.5-fold changes were determined by filtering to analyze miRNAs altered by the RH or ME49 strain of *Toxoplasma* infection.

### Transfection of miR-21 inhibitor

The cells were plated in six-well plates (3 × 10^5^ cells per well) in opti-MEM media (Gibco, Carlsbad, CA, USA) and were transfected with either miR-21 inhibitors using Lipofectamine 2000 (Invitrogen, Carlsbad, CA, USA). The transfected cells were cultured for 6 h, and the culture medium was then replaced with a fresh complete medium. The cells were harvested 24 h after the transfection.

### Statistical analysis

Statistical analyses were performed primarily using Student’s *t* test with Excel software (Microsoft, Washington DC, USA). For the analysis of animal experiments, a two-way analysis of variance with Prism 3.0 software was used (GraphPad Software, La Jolla, CA, USA). *P*-values < 0.05 were regarded as statistically significant.

### Ethics approval

All experimental protocols used in this study have been approved by the Institutional Review Board of Seoul National University College of Medicine (IRB no. SNU-150715-2).

## Supplementary Information

Supplementary Figures.

## Data Availability

The data used in the current study are available from the first author (BK Jung; mulddang@snu.ac.kr) upon reasonable request. And the microarray data of the current study are deposited in the GEO database under the accession number GPL32397.
