# Supplementary material for: Exosomal miRNA-21 from Toxoplasma gondii-infected microglial cells induces the growth of U87 glioma cells by inhibiting tumor suppressor genes
Source: Sci Rep. 2022 Sep 30;12:16450. doi: 10.1038/s41598-022-20281-w (PMC9525672; doi:10.1038/s41598-022-20281-w)
Supplement: Supplementary file 1 — Supplementary Figures. [file 41598_2022_20281_MOESM1_ESM.pdf]

Exosomal microRNA-21 from *Toxoplasma gondii*-infected microglial cells induces growth of U87 glioma cells by inhibiting tumor suppressor genes

Bong-Kwang Jung[1], Hyemi Song[1], Hyejoo Shin[1] & Jong-Yil Chai[1,2*]

[1]MediCheck Research Institute, Korea Association of Health Promotion, Seoul 07649, Korea.

[2]Department of Tropical Medicine and Parasitology, Seoul National University College of Medicine, Seoul 03080, Korea. Email: cjy@snu.ac.kr

[*]Corresponding author: Jong-Yil Chai (cjy@snu.ac.kr), Seoul National University College of Medicine, Seoul 03080, South Korea (Tel: +82-10-6269-3318).

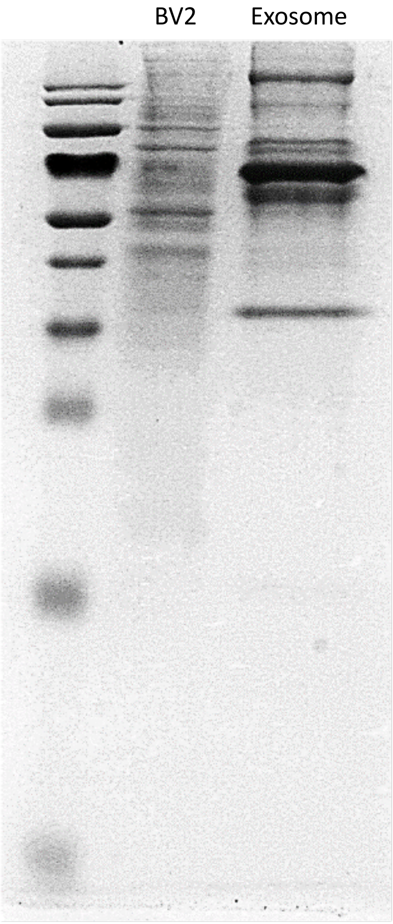

Figure S1. Characterization of BV2 cell-derived exosomes. The full image of SDS-PAGE. Protein was isolated from either BV2 cells or BV2-derived exosomes. The pattern of proteins was different between the cell and exosomes.

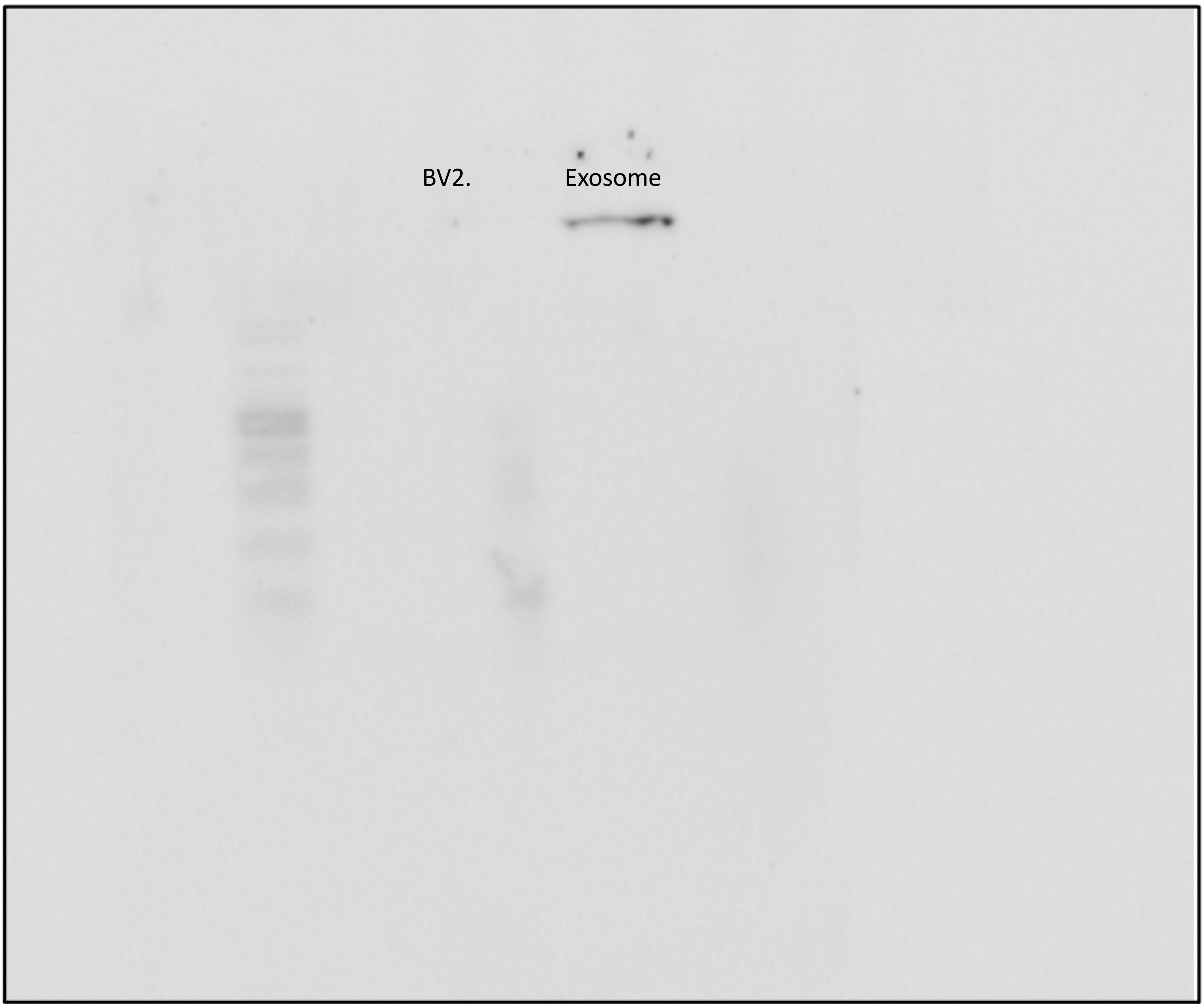

Figure S2. Characterization of BV2 cell-derived exosomes. The full image of western blot analysis of exosome marker (Alix).

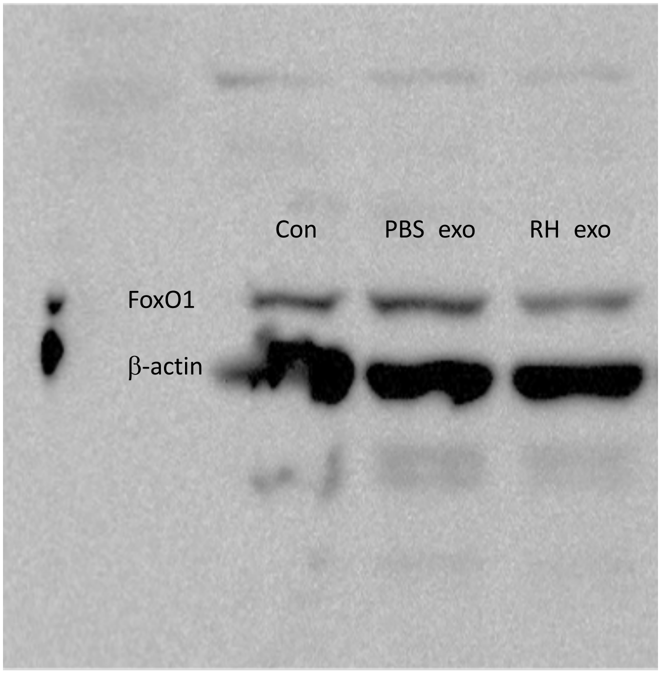

Figure S3. ***Toxoplasma*** **RH-infected BV2-derived exosomes induce downregulation of anti-tumor associated genes in U87 glioma cells by** ***Toxoplasma*** **RH-infected BV2-derived exosomes.** The full image of western blot analysis of FoxO1 expression.
